# Serum retinol binding protein 4 is negatively related to estrogen in Chinese women with obesity: a cross-sectional study

**DOI:** 10.1186/s12944-016-0215-6

**Published:** 2016-03-09

**Authors:** Qian Li, Weiyun Wu, Huandong Lin, Xinxia Chang, Hua Bian, Mingfeng Xia, Hongmei Yan, Xin Gao

**Affiliations:** Department of Endocrinology and Metabolism, Shanghai Zhongshan Hospital, Fudan University, Shanghai, 200032 China; Department of Clinical Laboratory, Shanghai Zhongshan Hospital, Fudan University, Shanghai, 200032 China

**Keywords:** Retinol binding protein 4, Estrogen, Obesity

## Abstract

**Background:**

The association between serum Retinol Binding Protein 4 (RBP4) and obesity is still controversial. Serum RBP4 levels varies by gender, and estradiol may play a role in the difference. To investigate the participation of sex hormones in the association of RBP4 and obesity in humans, we measured serum RBP4, BMI, and sex hormones in 87 women from the outpatient.

**Methods:**

Eighty-seven subjects of Chinese women origin from the outpatient (aged 40.22 ± 15.54 years) were enrolled. Subjects with diseases affecting the metabolic state or not suitable to participate in this study were excluded. Anthropometrics and laboratory tests, including lipid profile, luteinizing hormone (LH), follicle stimulating hormone (FSH), prolactin (PRL), estradiol (E2),progesterone (PROG), testosterone (TESTO), and dehydroepiandrosterone (DHEA) were conducted. Serum RBP4 was detected by an enzyme immunoassay kit and validated by quantitative Western blotting.

**Results:**

Circulating RBP4 levels were positively associated with BMI, waist circumference, waist-to-hip ratio (WHR), systolic and diastolic (SBP), diastolic blood pressure (DBP), triglycerides (TG), low high-density lipoprotein cholesterol(LDL-c), and testosterone (TESTO) in the total group. While only in obese individuals, serum RBP4 levels were negatively associated with E2. The highest value was in the subjects with both obesity and the low estrogen level. Multiple linear regression analysis revealed that RBP4 correlated independently with TG, TC and insulin in all subjects, TC in non- obese individuals. However, E2 were significantly associated with serum RBP4 only in obese individuals.

**Conclusions:**

RBP4 could be a marker of obesity-related factors; estrogen was negatively related to RBP4 and might be one of the influential factors.

## Background

Retinol-binding protein 4 (RBP4), belongs to the lipocalin family of proteins, which is released primarily by the liver, has also been shown to be released by adipose tissue as the second highest rate of expression [1, 2]. In humans, serum RBP4 levels have been suggested to provide the association with the degree of adiposity [3]. Obesity is associated with a dramatic increase in the prevalence of type 2 diabetes, and also has been associated with many other chronic diseases and comorbidities. Over the past few decades, the prevalence of obesity has increased greatly worldwide and in China, especially [4, 5]. Thus, the prevention of obesity among people should be considered a priority of health care policy.

Several studies show that serum RBP4 levels correlate inversely with the severity of insulin sensitivity, which is positively with obesity and type 2 diabetes, as well as components of the metabolic syndrome, and can be used as a noninvasive serological indexes reflecting the metabolic abnormalities [6–8]. However, there is also conflicting data between RBP4 and obesity was demonstrated in other studies [9, 10]. Therefore, the relationship of RBP4 and obesity is still controversial in adult. RBP4 does not reach adult plasma levels until puberty, after which plasma levels are changeable, based on the menstrual cycle. And this variation in the plasma RBP level appears to correlate with peak levels of estradiol and menopausal status in women [11, 12]. It also has been proved that plasma RBP4 concentration varies by gender, generally being lower in women than in men participants. Several previous studies hypothesized that sex hormones may play a role in the difference [10, 12, 13]. So it is necessary to investigate the participation of sex hormones on the levels of RBP4 in humans, especially in woman.

Therefore, the major aim of the present study is to evaluate the association between RBP4 levels, obesity, sex hormones, as well as other metabolic parameters in Chinese women. Additionally, we sought to explore the possible effect of sex hormones on the relation with obesity and RBP4.

## Methods

### Subjects

We consecutively selected 138 subjects from the general population who had undergone medical check-ups at the outpatient department of Endocrinology of Zhongshan hospital, Fudan university from Oct.2013 and Aug.2014. After excluding 51 of the 138 subjects, a total of 87 subjects were enrolled in our study. Exclusion factors were diseases affecting the metabolic state or not suitable to participate in this study. Women presenting endometriosis, uterinefibroid, breast cancer and those with hormone-dependent cancer were excluded. Hyperthyroidism, hypothyroidism, diabetes, mental disease, serious disease with dysfunction of heart, liver, kidney, were excluded as well as those using estrogen replacement therapy. The study was approved by the human research ethics committee of Zhongshan hospital, Fudan University and informed consent was obtained from all subjects.

### Anthropometric and biochemical measurements

Height (cm) and weight (kg) of participants, wearing light clothing and without shoes, was measured with stadiometers and calibrated balance-beam scales, respectively. BMI was calculated as weight in kilograms divided by the square of height in meters. Waist circumference (cm) was measured with a non-stretchable tape at the end of a normal expiration, at the smallest horizontal circumference between the ribs and iliac crest. Thus, waist–hip ratio (WHR) was calculated as waist circumference divided by hip circumference. Blood pressure was measured in the right arm in the seated position, after at least a 5 min rest, and the average of the three blood pressures was used as the final blood pressure. Blood samples were collected after a fasting period of at least 10 h overnight. Serum total cholesterol (TC), triglycerides (TG), high-density lipoprotein cholesterol (HDL-c), and low-density lipoprotein cholesterol (LDL-c) were determined by enzymatic methods with a Hitachi 7600 analyzer (Hitachi,Ltd. Tokyo, Japan). Serum insulin (mU/L) was measured with an insulin radioimmunoassay kit (kit from Beijing North Biotechnology Research Institute). Follicle stimulating hormone (FSH), luteinizing hormone (LH), prolactin (PRL), estradiol (E2), progesterone (PROG), testosterone (TESTO), dehydroepiandrosterone (DHEA) were checked. Methods for measuring blood hormones were electrochemiluminescence immunoassay (ECLIA), and kits were used for ECLIA supplied by Roche Diagnostic Systems, GmbH,Mannheim, Germany. Serum RBP4 (μg/ml) was measured in duplicate by a sandwich ELISA developed in-house, using affinity chromatography purified polyclonal and monoclonal antibodies generated against recombinant human RBP4 protein. The assay system was subsequently cross validated by Western blot analysis [14]. According to recommendations of the Working Group on Obesity in China, BMI between 18.5 and 24.0kg/m2 was considered normal. BMI between 24.0 and 28.0kg/m2 was defined as overweight, and over 28.0 was defined as obesity [15].

### Statistical analyses

Mean (standard deviation), median (interquartile range), were tabulated for demographic, and laboratory characteristics by BMI. Differences in the distributions of these variables between non-obese and obese were calculated by the Chi-Square test for categorical variables, ANOVA for normally distributed continuous variables. Following this, Pearson’s correlation was used to examine the association of serum RBP4 and other parameters. Multiple testing was corrected using LSD method (Equal Variances Assumed) or Games-Howell method (Equal Varance not assumed). Multiple stepwise regression analysis was used to examine the association of estrogen and serum RBP4 and other parameters. All statistical analyses were performed using SPSS18.0 (SPSS Inc., Chicago, Illinois, USA), *p* values <0.05 were considered significant.

## Results

The mean age of study participants was 40.22±15.54 years. Demographic and laboratory characteristics stratified by BMI are presented in Table 1. Compared to non-obese, obese individuals had higher waist circumference, hip circumference, waist-to-hip ratio (WHR), systolic and diastolic (SBP), diastolic blood pressure (DBP), triglycerides (TG), but lower high-density lipoprotein cholesterol (HDL-c), luteinizing hormone (LH), and follicle stimulating hormone (FSH). There are no differences between non-obese and obese individuals in age, total cholesterol (TC), low high-density lipoprotein cholesterol(LDL-c), prolactin (PRL), estradiol (E2), progesterone (PROG), testosterone (TESTO), dehydroepiandrosterone (DHEA), fasting insulin and serum RBP4.

**Table 1:** Basic characteristics of all the subjects

	Non-obese (*n* = 45)	Obese (*n* = 42)	*P* value
Age (year)	41.18 (16.07)	39.19 (15.08)	0.554
BMI (kg/m2)	22.69 (3.68)	34.79 (4.99)	<0.001
Waist (cm)	82.49 (10.55)	110.48 (14.53)	<0.001
Hip (cm)	94.09 (7.18)	116.65 (10.69)	<0.001
WHR (cm/cm)	0.88 (0.08)	0.95 (0.08)	<0.001
SBP (mmHg)	118.00 (16.67)	129.76 (16.7)	0.002
DBP (mmHg)	75.52 (8.55)	81.95 (11.92)	0.005
TC(mmol/L)	4.35 (0.94)	4.54 (0.83)	0.326
TG (mmol/L)	1.23 (0.70)	1.70 (0.90)	0.008
HDL-c (mmol/L)	1.27 (0.40)	1.02 (0.25)	0.001
LDL-c (mmol/L)	2.53 (0.81)	2.76 (0.70)	0.155
LH (mIU/mL)	8.00 (0.30–59.70)	5.70 (0.30–59.70)	0.018
FSH (mIU/mL)	5.60 (0.10–114.30)	5.30 (0.20–69.7)	0.046
PRL(mIU/mL)	380.60 (23.60–9544.00)	381.65 (10.40–2042.00)	0.562
E2 (pmol/L)	114.70 (22.20–617.60)	135.80 (37.20–859.90)	0.661
PROG (nmol/L)	1.40 (0.10–37.55)	1.40 (0.20–21.27)	0.733
TESTO (nmol/L)	0.99 (0.10–23.50)	1.78 (0.30–25.90)	0.510
DHEA (umol/L)	2.85 (0.40–16.70)	4.20 (1.20–18.50)	0.071
Fasting insulin (mU/L)	9.50 (5.00–57.20)	15.50 (3.60–60.50)	0.295
Serum RBP4 (μg/ml)	30.07 (7.83)	33.07 (7.06)	0.064

Data are presented as means(S.D) or median (range)

*BMI* body-mass index, *SBP* systolic blood pressure, *DBP* diastolic blood pressure, *WHR* waist-to-hip ratio, *TC* total cholesterol, *TG* triglycerides, *HDL-c* high-density lipoprotein cholesterol, *LDL-c* low-density lipoprotein cholesterol, *LH* luteinizing hormone, *FSH* folliclestimulating hormone, *PRL* prolactin, *E2* estradiol, *PROG* progesterone, *TESTO* testosterone, *DHEA* dehydroepiandrosterone, *RBP4* retinol binding protein 4

The Pearson correlation coefficients for associations between RBP4 concentrations and other parameters are summarized in Table 2. In all subjects, serum RBP4 levels were significantly positively associated with BMI, waist circumference, WHR, SBP, DBP, TC, TG, LDL-c and TESTO, but not associated with hip circumference, HDL-c, LH, FSH, PRL, E2, PROG and DHEA. In non-obese individuals, serum RBP4 levels were significantly positively associated with BMI, SBP, TC, TG, LDL-c, but not associated with waist circumference, hip circumference, DBP and the other variables mentioned above. However, in obese individuals, serum RBP4 levels were significantly positively associated with TC, TG, TESTO, and DHEA, whereas negatively associated with insulin and E2. But not associated with BMI, WHR and the other variables mentioned above, either.

**Table 2:** Pearson’s correlation analysis of serum RBP4 in all the subjects

	Total (*n* = 87)		Non-obese (*n* = 45)		Obese (*n* = 42)	
	*r*	*P*	*r*	*P*	*r*	*P*
BMI	0.308**	0.004	0.377*	0.011	0.158	0.316
Waist	0.258*	0.025	0.189	0.277	0.097	0.553
Hip	0.188	0.107	0.147	0.400	−0.070	0.666
WHR	0.271*	0.019	0.136	0.436	0.260	0.105
SBP	0.302**	0.005	0.352*	0.019	0.139	0.386
DBP	0.270*	0.013	0.276	0.069	0.198	0.214
TC	0.422**	*P* <0.001	0.467**	0.001	0.337*	0.029
TG	0.497**	*P* <0.001	0.315*	0.037	0.619**	*P* <0.001
HDL	−0.078	0.476	0.061	0.693	−0.129	0.415
LDL	0.295**	0.006	0.388**	0.009	0.115	0.468
LH	0.110	0.317	0.238	0.125	0.025	0.877
FSH	0.148	0.171	0.267	0.076	0.053	0.737
PRL	−0.151	0.166	−0.132	0.398	−0.255	0.103
E2	−0.207	0.113	−0.01	0.962	−0.374*	0.032
PROG	−0.119	0.298	−0.018	0.910	−0.317	0.063
TESTO	0.229*	0.045	0.102	0.541	0.344*	0.032
DHEA	0.210	0.055	0.050	0.747	0.329*	0.038
INSUL	−0.262	0.079	−0.258	0.418	−0.423*	0.013

*P <0.05; **P <0.01

*BMI* body-mass index, *SBP* systolic blood pressure, *DBP* diastolic blood pressure, *WHR* waist-to-hip ratio, *TC* total cholesterol, *TG* triglycerides, *HDL-c* high-density lipoprotein cholesterol, *LDL-c* low-density lipoprotein cholesterol, *LH* luteinizing hormone, *FSH* folliclestimulating hormone, *PRL* prolactin, *E2* estradiol, *PROG* progesterone, *TESTO* testosterone, *DHEA* dehydroepiandrosterone, *RBP4* retinol binding protein 4

**Fig. 1:**
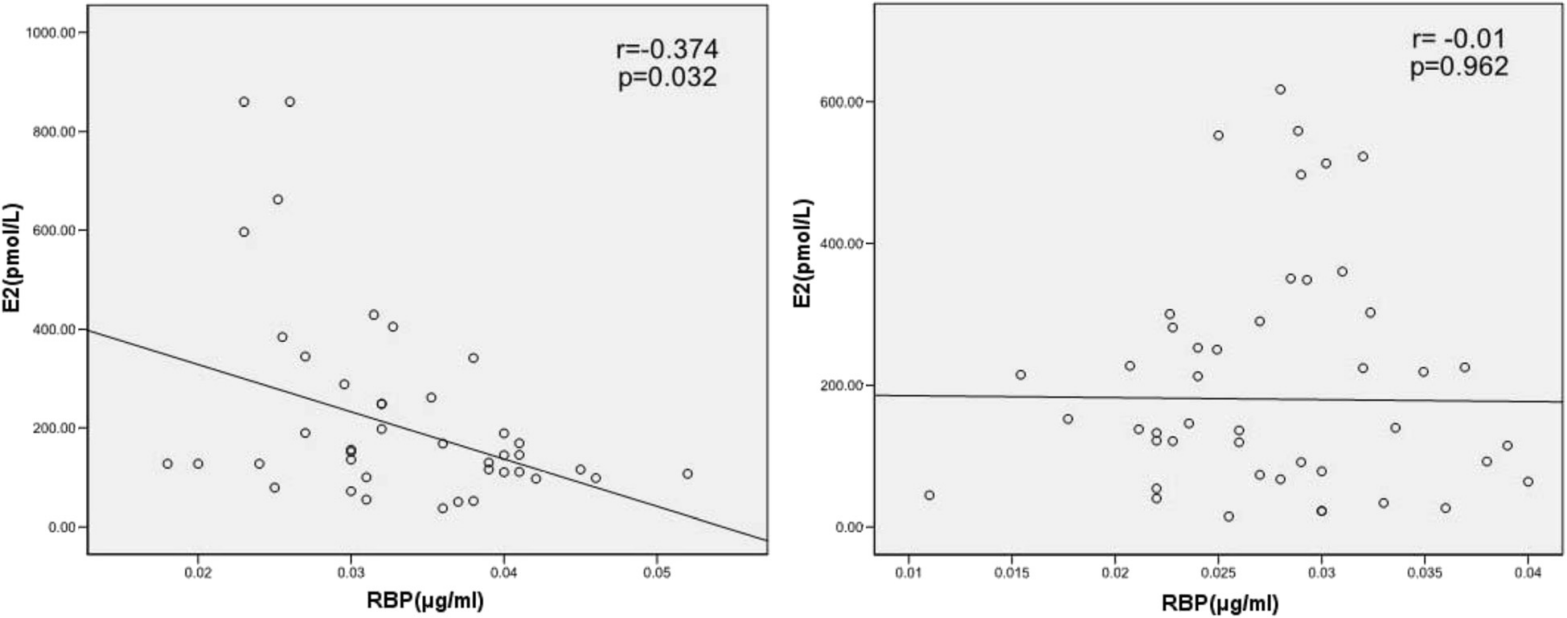
Pearson’s correlation analysis between RBP4 ((μg/ml)) and E2(pmol/L). Serum RBP4 level was negatively correlated with estrogen in obese individuals (*r* = −0.374, *p* = 0.032, **a** However, the association between RBP4 and estrogen no longer existed when in non-obese individuals (*r* = −0.01, *p* = 0.962, **b**)

Especially, we found that Serum RBP4 level was negatively correlated with estrogen in obese individuals (*r* = −0.374, *p* = 0.032), but no longer existed when in non-obese individuals (*r* = −0.01, *p* = 0.962) (Fig. 1). To further explore the association among serum RBP4 levels, BMI and E2, we built interaction terms between serum RBP4 levels and two categories of BMI and estradiol (E2) stratified by the median values at the same time. There were significant differences in serum RBP4 levels between groups classified by E2 level within the same BMI strata (Table 3). RBP4 levels were the highest in the subjects with both obesity and the low estrogen level (37.08 ± 7.29 μg/ml vs. 26.77 ± 3.47 μg/ml, 29.64 ± 7.8 μg/ml, and 30.9 ± 7.18 μg/ml, *P* for trend <0.001, respectively), compare to those with neither obesity nor low estrogen levels, individuals with no-obese but low estrogen levels, and individuals with obesity but high estrogen levels.

**Table 3:** The mean concentration of RBP4 in women stratified by BMI and estradiol (E2) levels

Non-obese		Obese	
E2 >118.60 (pmol/L)	E2 <118.60 (pmol/L)	E2 >118.60 (pmol/L)	E2 <118.60 (pmol/L)^a,b,c^
26.77 ± 3.47 (μg/ml)	29.64 ± 7.80 (μg/ml)	30.9 ± 7.18 (μg/ml)	37.08 ± 7.29 (μg/ml)

^a^ *P* <0.05 vs Non-obese group with high E2 level

^b^ *P* <0.05 vs Non-obese group with low E2 level

^c^ *P* <0.05 vs Obese group with high E2 level

E2 corresponds to baseline estradiol levels. Cut-off point was set at median value

A stepwise multiple linear regression analysis was performed using serum RBP4 level as a dependent variable. Independent variables were those parameters, which were significantly associated with RBP4 in Pearson’s correlation analysis. In all subjects, TG (Standardized Coefficients Beta = 0.509, *P* <0.001), TC (Standardized Coefficients Beta = 0.260, *P* = 0.047) together with Insulin (Standardized Coefficients Beta = −0.249, *p* = 0.035) were independent variables significantly associated with serum RBP4. In non-obese individuals, TC (Standardized Coefficients Beta = 0.582, *P* = 0.006) was independently associated with serum RBP4, while in obese individuals, Insulin (Standardized Coefficients Beta = −0.518, *p* = 0.006) together with E2 (Standardized Coefficients Beta = −0.438, *p* = 0.013) were significantly associated with serum RBP4 (Table 4).

**Table 4:** Multiple linear regression analysis of RBP4 and other parameters

		Standardized coefficients beta	t	Sig
ALL	TG	0.509	4.651	*P* <0.001
	TC	0.260	2.098	0.047
	INSUL	−0.249	−2.240	0.035
Non-obese	TC	0.582	3.113	0.006
Obese	E2	−0.438	−2.723	0.013
	INSUL	−0.518	−3.087	0.006

A step-wise multiple linear regression analysis was performed to determine the contributing factors to serum RBP4 levels. Independent variables included in multivariate regression model were variables which significantly associated with RBP4 in Pearson’s correlation analysis

## Discussion

In the current study, we found that serum RBP4 levels were inversely associated with E2 levels in a cohort of Chinese women patients with obesity. To the best of our knowledge, this study is the first showing that circulating RBP4 is associated not only with BMI but also E2 levels in Chinese women patients, which implied that estradiol might be an impact factor on the relation between obesity and circulating RBP4.

RBP4 has long-been known to be released by the liver, and it also has been shown that approximately 15% of circulating secretion results from adipose tissue, and down-regulates the glucose transporter GLUT4, which acts as the rate-limiting step in insulin-activated glucose transport across both muscle and adipocyte membranes [16, 17]. Results from the previous study also suggested that RBP4 levels had an important effect on obesity indices, as measured by BMI, WC, waist-to-hip ratio, and even body fat percentage [10, 14, 18, 19]. Especially, a prospective study suggested that baseline RBP4 levels predict subsequent increase in WC in a Korean adolescent population [20]. Similar to prior studies in adults, we found that the RBP4 levels were positively correlated with most of the obesity indices in Chinese women, including BMI, waist circumference, and WHR in the whole subjects. The results mentioned above indicate that RBP4 might be a useful marker of obesity-related factors in subjects. There are some discrepant results from other studies have questioned these associations, failing to demonstrate associations with obesity [21–23]. The possible reasons for this difference may due to different study subjects in the ethnicity, age, and size of the samples. However, when all women were divided into different groups according to BMI, there are no differences between non-obese and obese individuals in serum RBP4 levels. And the same link between circulating RBP4 levels and obesity indices was not founded in obese women, which implied that there are other mechanisms for regulating RBP4 levels.

In both adults and children, the concentration of serum RBP4 varies by gender, with levels lower in female than in male participants [13, 24, 25]. Several previous studies supported that RBP4 levels were shown to be different between premenopausal and postmenopausal women and vary according to menstrual cycle [11, 25, 26]. It hypothesized that sex hormones may play a role in the gender discrepancy, which may also plays protective effect on women against the influence of RBP4. In our result, we found that serum RBP4 levels were significantly negatively associated with estrogen (E2) only in obese women. When subjects were divided into four groups according to BMI and estrogen (E2) stratified by the median values, the highest value for serum RBP4 was in subjects with both obesity and the low estrogen level, the lowest value was in non-obese groups with high estrogen level. Multiple linear regression analysis also revealed that RBP4 correlated independently with TG, TC and insulin in all subjects, however, with E2 only in obese individuals. The possible mechanisms are as follow. Firstly, estrogen may have a direct effect on serum RBP4 expression. Tan et al found the RBP4 level is influenced by the effect of sex hormones and suggested 17b-estradiol have a direct regulatory effecting on the RBP4 concentration in women with polycystic ovary syndrome [27]. Other researches indicated estrogen mediates retinoic acid metabolism by the regulation of RBP4 expression in adipose tissue, and the systemic deficiency of serum estrogen may activate a specific regulatory process and consequently, which induce the overexpression of RBP4 through ER in visceral adipocytes [28, 29]. Secondly, estrogen may convey an indirect effect by influencing the body fat distribution on serum RBP4. As one kind of adipocytokines, RBP4 was identified a sexual dimorphism for secretion in previous studies, which was partly attributed to the effect of sex hormones and body fat distribution [30, 31]. Estrogen has an important role in regulation of fat metabolism, and the effect of estrogen to reduction of lipolysis was through activation of estrogen receptor alpha (ER-α) in adipose tissue, then influenced body adiposity [32–34]. Lack of estrogen, people tend to accrue more visceral fat, which has been highly correlated to metabolic and cardiovascular risk; whereas accrue less fat in the subcutaneous depot [35]. In our previous study, we have found that circulating RBP4 is positively associated with the visceral fat, which implied that visceral fat might be a major source of excess circulating RBP4 [36]. Consistent with our findings, relationship of RBP4 and visceral fat, not subcutaneous fat, was confirmed by many other studies in different populations [37–39], which suggest that visceral obesity might play a key role in increasing the circulating RBP4 level. Therefore, when combining several factors, the group with both obesity and low estrogen level has the highest serum RBP4 levels. We also found that testosterone was positively associated with RBP4 levels in women, for DHEA only in obese women.

The close relationship between other metabolic parameters and RBP4 in adult and adolescents has been observed in previous studies, in which RBP4 was positively associated with BP, lipid profiles, and FPG [20, 26, 40]. We also found that the RBP4 levels were positively correlated with most of the cardiometabolic risk factors, such as SBP, DBP, TC, TG and LDL. Especially, circulating RBP4 levels were found to be highly correlated with TC and TG, both in non-obese and obese subject. Consistent with our result, another research found higher triglyceride levels is correlated with higher RBP4 [41]. The possible mechanism is that retinoid have been known to affect the expression of several genes involved in triglyceride metabolism, including regulators of apo C-III production, hepatic and gastrointestinal triglyceride production and secretion, and beta-oxidation of fatty acids [42].

Some limitations of this study must be taken into consideration. Firstly, the cross-sectional design of the study represents a limitation, implicating that cause-and-effect relationship cannot be discerned. Secondly, our study subjects included only from the outpatient women. And the majority of individuals are middle-aged woman with variation of plasma estrogen, which may influence the levels of RBP4, as it changed during the menstrual cycle. The relation of RBP4 and estrogen is also restricted to only in obese women. Therefore, our results may not be applicable to the whole population. Finally, due to the limitation of data collection on all types of sex hormones, we got small sample size. As well as being small, the trials may have methodological weaknesses that could have resulted in bias.

## Conclusion

In conclusion, RBP4 could be a marker of obesity-related risk factors, and it is negatively related to estrogen, restricted to obese individuals. The RBP4 levels were higher in the co-presence of obesity and low estrogen, which suggested that their relationship was complicated and might influenced by estrogen and other confounding factors. Further experiments with larger groups of subjects are warranted to clarify the relationship between RBP4 and estrogen.

## Abbreviations

RBP4: retinol binding protein 4
BMI: body-mass index
SBP: systolic blood pressure
DBP: diastolic blood pressure
WHR: waist-to-hip ratio
TC: total cholesterol
TG: triglycerides
HDL-c: high-density lipoprotein cholesterol
LDL-c: low-density lipoprotein cholesterol
LH: luteinizing hormone
FSH: folliclestimulating hormone
PRL: prolactin
E2: estradiol
PROG: progesterone
TESTO: testosterone
DHEA: dehydroepiandrosterone
